# Survival After MI in a Community Cohort Study Contribution of Comorbidities in NSTEMI

**DOI:** 10.1016/j.gheart.2018.01.002

**Published:** 2018-03-05

**Authors:** Randi E. Foraker, Avirup Guha, Henry Chang, Emily C. O’Brien, Julie K. Bower, Elliott D. Crouser, Wayne D. Rosamond, Subha V. Raman

**Affiliations:** * Division of Cardiovascular Medicine, Ohio State University, Columbus, OH, USA; † Division of Epidemiology, College of Public Health, Ohio State University, Columbus, OH, USA; ‡ Davis Heart and Lung Research Institute, Ohio State University, Columbus, OH, USA; § Duke Clinical Research Institute, Durham, NC, USA; ‖ Division of Pulmonary, Allergy, Critical Care, and Sleep Medicine, Ohio State University, Columbus, OH, USA; ¶ Department of Epidemiology, University of North Carolina at Chapel Hill, Chapel Hill, NC, USA

## Abstract

**Background:**

Non–ST-segment elevation myocardial infarction (NSTEMI) comprises the majority of MI worldwide, yet mortality remains high. Management of NSTEMI is relatively delayed and heterogeneous compared with the “time is muscle” approach to ST-segment elevation MI, though it is unknown to what extent comorbid conditions drive NSTEMI mortality.

**Objectives:**

We sought to quantify mortality due to MI versus comorbid conditions in patients with NSTEMI.

**Methods:**

Participants of the ARIC (Atherosclerosis Risk in Communities) study cohort ages 45 to 64 years, who developed incident NSTEMI were identified and incidence-density matched to participants who did not experience an MI by age group, sex, race, and study community. We estimated hazard ratios for all-cause mortality, comparing those who developed NSTEMI to those who did not experience an MI.

**Results:**

ARIC participants with incident NSTEMI were more likely at baseline to be smokers, have diabetes and renal dysfunction, and take blood pressure or cholesterol-lowering medications than were participants who did not have an MI. Over one-half of participants experiencing NSTEMI died over a median follow-up of 8.4 years; incident NSTEMI was associated with 30% higher risk of mortality after adjusting for comorbid conditions (hazard ratio: 1.30; 95% confidence interval: 1.11 to 1.53).

**Conclusions:**

NSTEMI confers a significantly higher mortality hazard beyond what can be attributed to comorbid conditions. More consistent and effective strategies are needed to reduce mortality in NSTEMI amid comorbid conditions.

Ischemic heart disease, often manifest as myocardial infarction (MI), is the leading cause of disease burden worldwide [1]. MI are typically categorized as either ST-segment elevation (STEMI) or non–ST-segment elevation (NSTEMI) based on electrocardiographic (ECG) findings, the latter comprising approximately 70% of all MI [2]. Guidelines strongly endorse invasive management for patients with STEMI within 120 min of first medical contact based on considerable evidence that early reperfusion reduces myocardial damage and improves outcomes [3]. Conversely, guidelines for patients with NSTEMI advocate an “early” invasive strategy that includes angiography at some time within 24 h of presentation [4]. This approach may improve outcomes in the highest risk subset of the heterogeneous NSTEMI population, though paradoxically this strategy is most consistently applied to lower risk patients with NSTEMI where benefit remains uncertain [5,6].

Despite similar or greater mortality in patients with NSTEMI, their risk compared with that of patients with STEMI is typically perceived by health care providers as lower [7]. Furthermore, worse outcomes in NSTEMI are often attributed to comorbid conditions such as diabetes [8], impaired renal function [9], and lung disease [10]. Underestimation of risk and attribution of risk to comorbidities rather than the MI itself may explain why coronary angiography and revascularization, part of the standard of care in STEMI, are performed much less expediently in NSTEMI [11,12], a practice that may compromise myocardial health and contribute to poor outcomes.

To date, a direct comparison of survival in NSTEMI versus a suitable comparison population without MI but accounting for comorbidities has not been performed. Such a survival comparison could better define the importance of time-sensitive, myocardium-directed management in NSTEMI. In this work, we analyzed data from a community-based cohort study to test the hypothesis that the hazard of all-cause mortality, controlling for the effects of participant characteristics and common comorbidities, would be higher among those experiencing NSTEMI than among those without an MI.

## METHODS

The ARIC (Atherosclerosis Risk in Communities) study cohort was recruited beginning in 1987, during which time each ARIC field center (suburbs of Minneapolis, Minnesota; Washington County, Maryland; Forsyth County, North Carolina; and Jackson, Mississippi) enrolled a sample of approximately 4,000 individuals ages 45 to 64 years. ARIC methodology is described in detail elsewhere [13]; briefly, a total of 15,792 participants had an extensive baseline examination, including medical, social, and demographic data collection. These participants were re-examined every 3 years from 1990 to 1992, 1993 to 1995, and 1996 to 1998, respectively. Follow-up still occurs yearly by telephone to maintain contact with participants. Institutional review boards approved the study protocols, and all participants provided informed consent. Time to death was the outcome of interest for these analyses. Vital status of ARIC participants was ascertained as part of aforementioned annual follow-up and also confirmed by death certificate, hospitalization record, or next-of kin or physician report [13].

For our primary analysis, the exposed series comprised participants who experienced an NSTEMI during follow-up. Those who did not experience an MI composed the unexposed series of participants. NSTEMI classification in the ARIC study was based on a hospital discharge diagnosis of probable or definite MI, an equivocal or abnormal cardiac biomarker, and lack of ST-segment elevation.

After excluding 647 participants with prevalent MI at baseline, we excluded an additional 192 participants because of insufficient numbers for analyses, such as race/ethnicity other than white or black, along with participants with incident STEMI during follow-up, resulting in a final sample size of 422 participants with NSTEMI during the follow-up period. Among 14,459 eligible participants, 14,037 did not have an incident NSTEMI through 2011 and were eligible for inclusion in the unexposed series. Selecting from the unexposed series with replacement participants without an MI were successfully incidence-density matched to NSTEMI cases as described in detail in the Statistical Analysis section.

Covariates measured at baseline included age, sex, race, and study community; along with selected socioeconomic, clinical, and behavioral characteristics as follows. Common and clinically significant comorbidities were selected a priori for consideration in the analyses. Body mass index (kg/m^2^) and creatinine (mg/dl) were measured during the medical exam at baseline and were treated continuously in all analyses. Educational attainment was self-reported at baseline and categorized as less than high school or high school or greater. Smoking status was self-reported at baseline and defined as current, former, or never. Medical history was determined via self-report and medical exam at baseline for cancer, diabetes, and lung disease. The presence of left ventricular hypertrophy (LVH) was determined from ECG data. ECG-LVH was defined 2 ways: 1) by Minnesota Codes (3-1 or 3-3) and (any of 4-1 to 4-3 or 5-1 to 5-3), which represent high-amplitude left chest R waves and ST-T changes; and 2) by Cornell voltage criteria. Participants were described as currently taking aspirin, blood pressure–lowering medication, or cholesterol-lowering medications if they reported use within 2 weeks of the baseline exam.

### Statistical analyses

Exposed and unexposed series were matched using an incidence-density matching strategy on 5-year age group at baseline (45 to 49, 50 to 54, 55 to 59, and 60 to 64 years), sex, and race/study community (whites living in Minnesota, Maryland, or North Carolina, or blacks living in North Carolina or Mississippi) at the time the NSTEMI occurred. Up to 5 participants were matched to each NSTEMI case based on these factors for a total of 608 participants in the unexposed series. All analyses accounted for the matched structure of the data.

Survival was assessed from the time of the MI event to death, loss to follow-up, or the end of 2011, whichever came first. Consistent with the incidence-density sampling strategy, the follow-up for the unexposed group began on the same calendar date as did the follow-up for their matched NSTEMI counterparts. We calculated the median time-to-event for each exposure group. The product-limit (Kaplan-Meier) method was used to measure time to death over the course of follow-up. We performed matched Cox proportional hazards regression (frailty models) to estimate hazard ratios (HR) and 95% confidence intervals (95% CI) for all-cause mortality, comparing participants with NSTEMI to the unexposed series. This modeling strategy accounted for the dependence of observations induced by matching.

Crude NSTEMI-mortality analyses were conducted, and the influence of covariates were tested in a full multivariable model. Model variable selection was assessed using a p value of <0.05. Analyses were performed with SAS version 9.3 (SAS Institute Inc., Cary, North Carolina) statistical software.

### Secondary analyses

We additionally assessed for the effect of NSTEMI, on mortality risk, conditional on surviving 30 days following the MI event. According to these criteria, we included 381 participants with NSTEMI in the exposed group and 185 in the unexposed group.

## RESULTS

### NSTEMI

Table 1 compares the baseline characteristics of ARIC participants who developed NSTEMI to those of participants who did not have an MI over the course of follow-up. Participants who experienced NSTEMI over follow-up had a statistically significantly higher prevalence of diabetes (25.4% vs. 12.9%), current smoking (42.2% vs. 25.8%), and LVH (5.2% vs. 2.2%) at baseline compared with participants who did not experience an MI.

**FIGURE 1:**
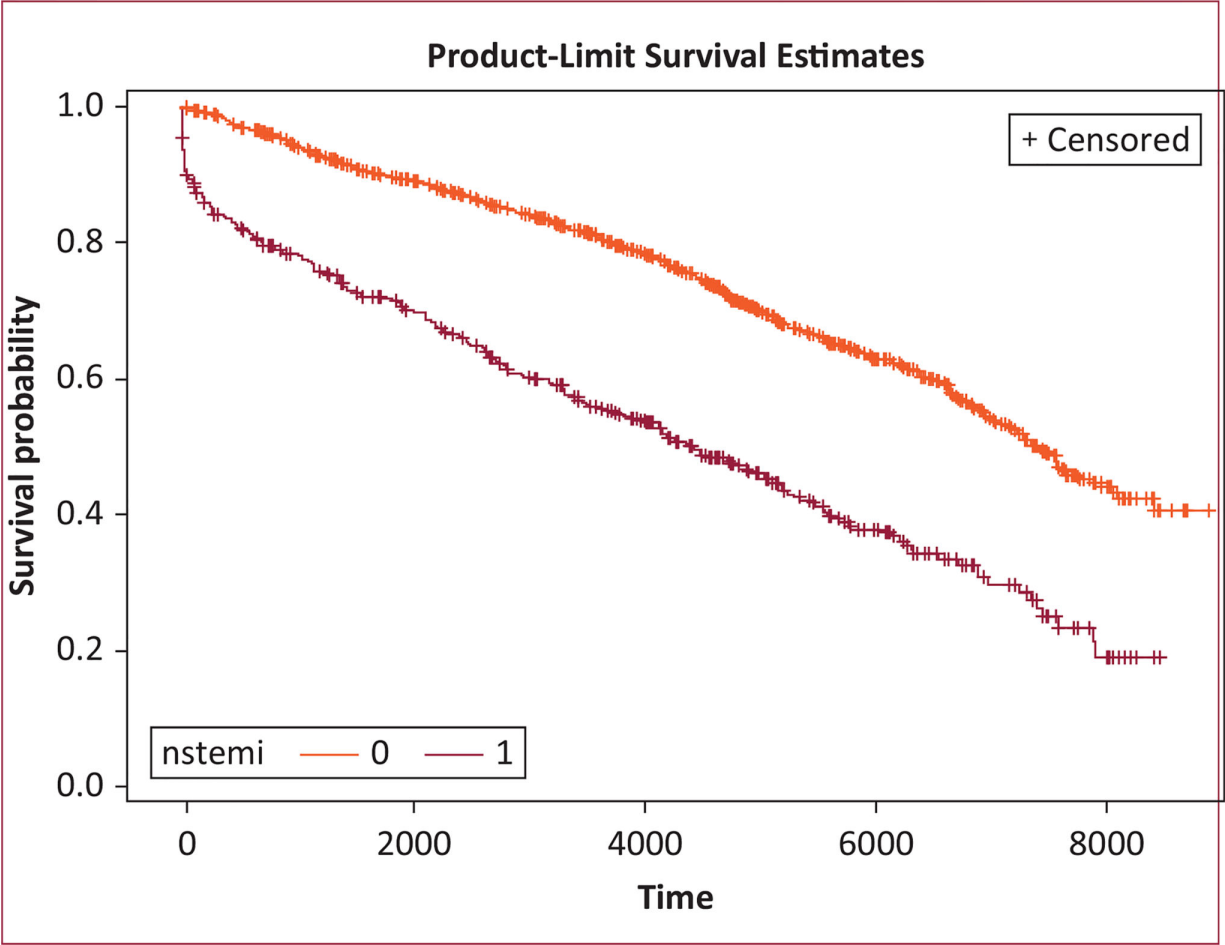
Kaplan-Meier survival curves demonstrating a difference in survival between non–ST-segment elevation myocardial infarction (NSTEMI) (red) and myocardial infarction–free (orange) participants from the ARIC (Atherosclerosis Risk in Communities) study. Time is shown in days.

Of the 422 participants who developed NSTEMI, 56% (n = 238) died over a median follow-up of 8.4 years. In contrast, among those who did not experience an MI, 74 (40%) died over a median 9.4 years of follow-up. Kaplan-Meier curves demonstrate a survival difference between NSTEMI and MI-free groups, with the MI-free group experiencing a lower hazard of mortality over the follow-up period (log-rank p < 0.0001) (Figure 1).

Table 2 presents minimally adjusted (model 1) and fully adjusted (model 2) HR and 95% CI. Adjusting for age, sex, and race/study community, NSTEMI was associated with an increased hazard of mortality (HR: 1.66; 95% CI: 1.44 to 1.93) compared with participants without an MI. Clinical factors measured at baseline that were significantly associated with mortality risk after adjustment for age, sex, and race/study community were creatinine, smoking status, history of cancer and LVH, and those taking aspirin and blood pressure medication (Table 2). In sensitivity analyses, the effect of NSTEMI remained after conditioning on survival to 30 days (HR: 1.37; 95% CI: 1.17 to 1.60).

In the fully adjusted model, the majority of the aforementioned associations remained but were slightly attenuated (Table 2); the NSTEMI group remained at a higher mortality risk (HR: 1.30; 95% CI: 1.11 to 1.53) compared with the MI-free group. Baseline factors that maintained statistical significance in the fully adjusted multivariable model were creatinine, smoking status, history of cancer and LVH, and those taking aspirin and blood pressure–lowering medication. History of diabetes was statistically significantly associated with the hazard of mortality in the multivariable model (Table 2).

## DISCUSSION

Community participants experiencing incident NSTEMI had a higher hazard of mortality than participants who were MI-free. Whereas those with NSTEMI events had a higher proportion of comorbidities and adverse health behaviors at baseline, NSTEMI itself remained a significant risk factor for mortality after adjustment for these conditions, and this difference persisted over the follow-up period and in analyses conditional on 30-day survival.

It may seem obvious that experiencing NSTEMI should confer greater subsequent risk of death compared with not having an MI. However, because this MI-associated mortality hazard persisted despite adjustment for comorbid conditions, myocardial damage itself—the essence of an MI—becomes the lead suspect responsible for downstream mortality. One could then intuit that strategies that reduce myocardial damage in patients with MI should be evaluated to reduce mortality. This logic has translated to remarkably consistent deployment of myocardial damage-limiting approaches for patients with STEMI. For these patients, the adage “time is muscle” has driven changes in health care delivery such as engagement between rural emergency medical services and interventional cardiologists, in-ambulance ECG findings that drive upstream drug delivery, and 24/7/365 in-house staffing of major cardiac catheterization laboratories.

Although the study does not directly compare management strategies or MI types, strategies that reduce myocardial damage and ultimately lower mortality in other conditions may warrant evaluation for patients with NSTEMI. A recently published randomized trial for patients with NSTEMI showed lower 30-day death and myocardial damage with immediate (within an average of 1.4 h) versus delayed (median: 61.0 h) angiography [14]. Although further evaluation of these provocative findings is needed, they do support a potential mortality benefit via timely interventions that limit damage to at-risk myocardium in NSTEMI. We also showed that the mortality curves separate early from MI-free participants in NSTEMI. This highlights the point of “time is muscle” with possibly more acute ischemia-driven death that might be easily treatable by early revascularization compared with delayed outcomes of like heart failure or scar related arrhythmia. Similar findings of a benefit for short-term efficacy of early intervention have been shown in the GUSTO (Global Use of Strategies to Open Occluded Coronary Arteries) IIa study [15] and the ARIC community surveillance study as well [16]. As blood biomarkers and ECG at time of presentation with NSTEMI may be equivocal, delaying diagnosis and treatment, direct identification of myocardium at risk with edema imaging, or other approaches may help ensure that patients with NSTEMI who have at-risk but salvageable myocardium similar to patients with STEMI get more timely invasive assessment than what current practice allows.

Whereas prior studies have shown that comorbidities such as diabetes [8], chronic kidney disease [9], and lung disease [10] increase MI mortality, none have evaluated the incremental mortality hazard of the MI itself while holding these common comorbidities constant. This is an important distinction, particularly when contemporary cardiovascular practice views patients with NSTEMI as burdened with significant comorbidities that may influence urgency of invasive assessment. Yusuf et al. [17] showed improved survival in multivariable analysis of patients with cancer suffering MI with guideline-based medical therapy; they also demonstrated a trend toward improved survival with revascularization, though use of percutaneous coronary intervention was remarkably low (3.3%) in this cohort.

Similarly, patients with chronic kidney disease suffering NSTEMI receive less guideline-directed therapy [18,19] than do patients with chronic lung disease and NSTEMI [20]. Even in the presence of those and other important covariates that make individuals more susceptible to mortality, participants with NSTEMI experienced worse outcomes.

### Study limitations

Even though our work has many strengths, particularly compared with claims data–based approaches, including careful classification matching of patients with and without NSTEMI to an MI-free population and participant diversity, limitations include reliance on baseline comorbidity information that may have changed by the time of incident NSTEMI in those experiencing MI events. Further investigations including patients whose race and ethnicity extend beyond black and white communities are needed. There remains potential for residual confounding in these analyses that likely biases the observed effect estimates up and away from the null. We also cannot comment on changes in medication between the event and follow-up, which would influence mortality. As there were no systematic cardiac imaging or functional status assessments post-MI, further risk stratification of the post-MI cohort by such data as wall motion abnormalities or heart failure was not feasible. This study spans a significant time period that provides strength to the data in terms of follow-up length; however, the study also spans generations of various different therapies of ischemic heart disease. These limitations extend to both participants with MI and their matched referent group.

## CONCLUSIONS

We have shown that patients with NSTEMI, where comorbid conditions are common, have increased risk of death beyond what can be accounted for by these comorbidities. Further studies of strategies that limit myocardial damage, the central feature of MI, to reduce mortality in patients with NSTEMI are warranted.

## Figures and Tables

**TABLE 1:** Baseline characteristics of eligible ARIC cohort participants by follow-up NSTEMI status

	NSTEMI (n = 422)	MI-Free (n = 186)	p Value
Age, yrs	55.4 ± 5.4	54.7 ± 5.9	N/A

Sex			
Female	119 (47.2)	94 (50.5)	N/A
Male	223 (52.8)	92 (49.5)	

Race/study community			
Black/MS	100 (23.7)	39 (21.0)	N/A
Black/NC	12 (2.8)	29 (15.5)	
White/MD	109 (25.8)	40 (21.5)	
White/MN	89 (21.2)	39 (21.0)	
White/NC	112 (26.5)	39 (21.0)	

Body mass index, kg/m^2^	28.8 ± 5.2	28.0 ± 5.7	0.004

Creatinine, mg/dl	1.2 ± 0.7	1.1 ± 0.2	0.9

Education			
Less than high school	290 (68.7)	129 (69.4)	0.3
High school or greater	132 (31.3)	57 (30.6)	

Smoking status			
Current	178 (42.2)	48 (25.8)	<0.0001
Former	121 (28.7)	56 (30.1)	
Never	123 (29.1)	82 (44.1)	

Cancer			
Yes	26 (6.2)	12 (6.5)	0.8
No	396 (93.8)	174 (93.5)	

Diabetes			
Yes	107 (25.4)	24 (12.9)	0.003
No	315 (74.6)	162 (87.1)	

Left ventricular hypertrophy			
Yes	22 (5.2)	4 (2.2)	<0.0001
No	400 (94.8)	182 (97.8)	

Lung disease			
Yes	26 (6.2)	9 (4.8)	0.7
No	396 (93.8)	177 (95.2)	

Aspirin			
Yes	199 (47.2)	84 (45.2)	0.5
No	223 (52.8)	102 (54.8)	

Blood pressure-lowering medication			
Yes	161 (38.2)	56 (30.1)	0.01
No	261 (61.8)	130 (69.9)	

Cholesterol-lowering medication			
Yes	20 (4.7)	0 (0)	0.009
No	402 (95.3)	186 (100)	

Deceased			
Yes	238 (56.4)	74 (39.8)	0.0002
No	184 (43.6)	112 (60.2)	

Follow-up time, days, median	3,074.5	3,416.0	N/A

Values are mean ± SD or n (%), unless otherwise indicated.

ARIC, Atherosclerosis Risk in Communities; MD, Maryland; MN, Minnesota; MS, Mississippi; N/A, not applicable; NC, North Carolina; NSTEMI, non–ST-segment elevation myocardial infarction.

**TABLE 2:** Minimally adjusted and fully adjusted HR and 95% CI for mortality among ARIC cohort participants with and without NSTEMI

	Model 1*	Model 2†
NSTEMI		
Yes	1.66 (1.44–1.93)	1.30 (1.11–1.53)
No	Ref.	Ref.

Age, yrs		
60–64	4.05 (3.13–5.23)	2.87 (2.35–3.52)
55–59	2.21 (1.80–2.72)	2.68 (2.23–3.23)
50–54	2.21 (1.85–2.64)	2.33 (2.00–2.71)
45–49	Ref.	Ref.

Sex		
Male	0.90 (0.77–1.05)	0.98 (0.85–1.12)
Female	Ref.	Ref.

Race/study community		
Black/NC	1.79 (1.43–2.23)	1.24 (0.99–1.55)
Black/MS	1.35 (1.11–1.65)	1.02 (0.82–1.27)
White/MN	1.34 (0.84–2.15)	1.22 (0.81–1.84)
White/NC	1.70 (1.38–2.08)	1.29 (1.05–1.58)
White/MD	Ref.	Ref.

Body mass index, kg/m^2^		
1-unit change	1.00 (0.99–1.00)	1.00 (0.99–1.00)

Creatinine, mg/dl		
1-unit change	1.40 (1.30–1.51)	1.31 (1.22–1.40)

Education		
Less than high school	1.08 (0.95–1.23)	0.94 (0.82–1.08)
High school or greater	Ref.	Ref.

Smoking status		
Current	2.96 (2.52–3.49)	2.53 (2.14–2.99)
Former	2.79 (2.41–3.23)	2.32 (1.98–2.72)
Never	Ref.	Ref.

Cancer		
Yes	2.22 (1.81–2.72)	1.51 (1.20–1.90)
No	Ref.	Ref.

Diabetes		
Yes	0.58 (0.50–0.68)	1.41 (1.20–1.67)
No	Ref.	Ref.

Left ventricular hypertrophy		
Yes	2.14 (1.61–2.84)	1.29 (0.92–1.80)
No	Ref.	Ref.

Lung disease		
Yes	0.96 (0.73–1.28)	0.93 (0.71–1.23)
No	Ref.	Ref.

Aspirin		
Yes	1.20 (1.06–1.37)	1.04 (0.92–1.19)
No	Ref.	Ref.

Blood pressure-lowering medication		
Yes	1.57 (1.38–1.78)	1.35 (1.17–1.57)
No	Ref.	Ref.

Cholesterol-lowering medication		
Yes	1.65 (0.96–2.84)	1.02 (0.59–1.77)
No	Ref.	Ref.

CI, confidence interval; HR, hazard ratio; other abbreviations as in Table 1.

* Model 1: Each variable was run in a separate model, controlling for age, sex, and race.

† Model 2: Results of the full model, controlling for age, sex, race, plus all other covariates.
